# Blockade of Serotonin 5-HT_6_ Receptor Constitutive Activity Alleviates Cognitive Deficits in a Preclinical Model of Neurofibromatosis Type 1

**DOI:** 10.3390/ijms221810178

**Published:** 2021-09-21

**Authors:** Emilie Doucet, Katarzyna Grychowska, Pawel Zajdel, Joël Bockaert, Philippe Marin, Carine Bécamel

**Affiliations:** 1 Institute of Functional Genomics, University of Montpellier, CNRS, Inserm, 34094 Montpellier, France; emilie.l.doucet@gmail.com (E.D.); joel.bockaert@igf.cnrs.fr (J.B.); 2 Department of Organic Chemistry, Jagiellonian University Medical College, 9 Medyczna Street, 30-688 Kraków, Poland; katarzyna.grychowska@uj.edu.pl (K.G.); pawel.zajdel@uj.edu.pl (P.Z.)

**Keywords:** neurofibromatosis type 1, 5-HT_6_ receptor, constitutive activity, inverse agonist, neutral antagonist, mTOR, rapamycin, cognition

## Abstract

Neurofibromatosis type 1 (NF1) is a common inherited disorder caused by mutations of the *NF1* gene that encodes the Ras-GTPase activating protein neurofibromin, leading to overactivation of Ras-dependent signaling pathways such as the mTOR pathway. It is often characterized by a broad range of cognitive symptoms that are currently untreated. The serotonin 5-HT_6_ receptor is a potentially relevant target in view of its ability to associate with neurofibromin and to engage the mTOR pathway to compromise cognition in several cognitive impairment paradigms. Here, we show that constitutively active 5-HT_6_ receptors contribute to increased mTOR activity in the brain of *Nf1^+/−^* mice, a preclinical model recapitulating some behavioral alterations of NF1. Correspondingly, peripheral administration of SB258585, a 5-HT_6_ receptor inverse agonist, or rapamycin, abolished deficits in long-term social and associative memories in *Nf1^+/−^* mice, whereas administration of CPPQ, a neutral antagonist, did not produce cognitive improvement. These results show a key influence of mTOR activation by constitutively active 5-HT_6_ receptors in NF1 cognitive symptoms. They provide a proof of concept that 5-HT_6_ receptor inverse agonists already in clinical development as symptomatic treatments to reduce cognitive decline in dementia and psychoses, might be repurposed as therapies alleviating cognitive deficits in NF1 patients.

## 1. Introduction

Neurofibromatosis type 1 (NF1) is a dominant autosomal disease with an estimated prevalence of about 1 in 3,000 that is independent of ethnicity, race, or gender, and with a full penetrance. The hallmarks of the disease are “café au lait” spots and tumors of central and peripheral nervous systems, including neurofibromas, gliomas and pheochromocytomas [1]. Cognitive deficits represent another major feature of the disease, with up to 80% of the children with NF1 at risk of moderate to severe cognitive impairments that affect one or more areas of cognitive functioning and seriously compromise their scholar performance and quality of life [2,3]. These include learning disability, decreased attention, difficulties in executive planning and deficits in perception skills [4,5,6]. Moreover, at least half of children and adults with NF1 show social behavior impairments [7,8,9,10]. To date, clinical trials assessing the effect of statins or methylphenidate upon behavioral and cognitive outcomes yielded mitigated or poorly reproducible results [11,12,13,14,15], underscoring the need of new therapeutic strategies.

NF1 is caused by heterozygous mutations of the *NF1* tumor suppressor gene that encodes the Ras-GTPase activating protein (Ras-GAP) neurofibromin. Reduced expression of neurofibromin or loss of function mutations of the *NF1* gene lead to overactivation of Ras that results in aberrant stimulation of Ras-dependent signaling pathways such as the Raf-MEK-Erk and mechanistic Target of Rapamycin (mTOR) pathways [16,17,18,19]. Previous studies have shown that neurofibromin physically interacts with and promotes constitutive activity of the serotonin 5-HT_6_ receptor [20,21], a Gs-coupled receptor predominantly expressed in brain regions involved in cognitive functions such as the prefrontal cortex (PFC) and exhibiting a high level of constitutive activity. In addition to its coupling to Gs protein, the 5-HT_6_ receptor also engages mTOR signaling to compromise cognition in neurodevelopmental models of schizophrenia [20] and preclinical models of cannabis abuse during adolescence [22] and neuropathic pain [23]. Conversely, reduced 5-HT_6_ receptor-operated mTOR signaling has been involved in memory enhancement elicited by dietary restriction in the mouse [24]. Collectively, these findings suggest that blocking the 5-HT_6_ receptor-mTOR pathway might be a promising strategy to alleviate cognitive deficits associated with neuropsychiatric disorders of different etiologies [20,22,25,26,27].

In light of these observations and the previously established physical and functional interactions between the 5-HT_6_ receptor and neurofibromin, we investigated the impact of inhibiting 5-HT_6_ receptor or mTOR activation on social cognition and associative memory in mice with a heterozygous null mutation in the *NF1* gene (*Nf1^+/−^* mice), a well-characterized preclinical model of NF1 that recapitulates some behavioral features of the disease [28,29,30,31,32]. We show that the acute injection of SB258585, a 5-HT_6_ receptor antagonist that behaves as inverse agonist [23], or of rapamycin, a mTOR inhibitor, abolishes deficits in long-term social and associative memories in *Nf1^+/−^* mice and reduces mTOR overactivation in the PFC of these mice. Conversely, administration of (*S*)-1-[(3-chlorophenyl)sulfonyl]-4-(pyrrolidine-3-yl-amino)-1*H*-pyrrolo [3,2-c]quinolone (CPPQ), a 5-HT_6_ receptor neutral antagonist [21,33,34], does not induce any cognitive improvement, suggesting that mTOR under the control of constitutively active 5-HT_6_ receptors, contributes to cognitive symptoms of NF1.

## 2. Results

### 2.1. Role of 5-HT_6_ Receptor in mTOR Overactivation in Prefrontal Cortex of Nf1^+/−^ Mice

**Figure 1:**
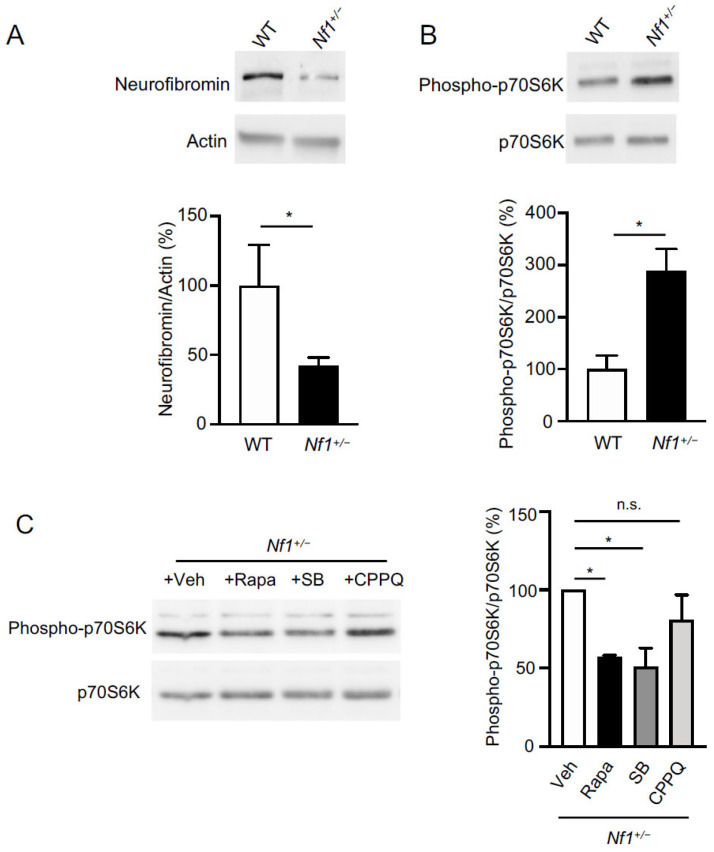
Role of constitutively active 5-HT_6_ receptors in increased mTOR activity in prefrontal cortex of *Nf1^+/−^* mice. (**A**). Representative Western blot assessing neurofibromin expression in PFC of adult WT and *Nf1^+/−^* mice. Data represent the ratios of immunoreactive signals of the anti-neurofibromin antibody to the immunoreactive signal of the anti-β-actin antibody and are expressed in % of values in WT mice. (**B**). Representative Western blots assessing p70S6K phosphorylation at Thr^421^-Ser^424^ as an index of mTOR activity in PFC of adult WT and *Nf1^+/−^* mice. Data represent the ratios of immunoreactive signals of the anti-phospho-Thr^421^-Ser^424^-p70S6K antibody to the immunoreactive signal of the anti-p70S6K antibody and are expressed in % of values in WT mice. In (**A**,**B**), the data illustrated are the mean ± SEM of results obtained in three mice for the WT genotype and five mice for the *Nf1^+/−^* genotype. * *p* < 0.05, unpaired *t*-test. (**C**). Western blots assessing p70S6K phosphorylation at Thr^421^-Ser^424^ in PFC of *Nf1^+/−^* mice injected with either vehicle (*n* = 3) or Rapamycin (Rapa, 10 mg/kg, *n* = 3), or SB258585 (SB, 2.5 mg/kg, *n* = 3) or CPPQ (2.5 mg/kg, *n* = 3) 30 min before sacrifice. Data are expressed as in B. * *p* < 0.05, one-way ANOVA followed by Dunnett’s test. n.s.: not significant.

Corroborating previous observations [21,28], a ~50% reduction of neurofibromin expression was measured in the PFC of *Nf1^+/−^* mice, compared to wildtype (WT) mice (Figure 1A, see also Supplementary Figure S1A for raw data). As expected, this reduced expression of neurofibromin was accompanied with an increase in the mTOR activity in PFC, as assessed by the enhanced phosphorylation state of its substrate 70 kDa ribosomal protein S6 kinase (p70S6K) on Thr^421^-Ser^424^ (Figure 1B, see also Supplementary Figure S1B for raw data), which was abolished by an acute administration of rapamycin (10 mg/kg i.p., 30 min before the sacrifice, Figure 1C, see also Supplementary Figure S1C for raw data). Administration of the 5-HT_6_ receptor inverse agonist SB258585 (2.5 mg/kg i.p., 30 min) strongly reduced the enhanced phosphorylation of p70S6K on Thr^421^-Ser^424^, thereby reproducing the effect of rapamycin treatment (Figure 1C). In contrast, administration of the 5-HT_6_ receptor neutral antagonist CPPQ (2.5 mg/kg i.p., 30 min) did not diminish the enhanced mTOR activation in the PFC of *Nf1^+/−^* mice (Figure 1C).

### 2.2. Effect of Blocking the 5-HT_6_ Receptor-mTOR Pathway on Sociability and Short-Term and Long-Term Social Memories of Nf1^+/−^ Mice

**Figure 2:**
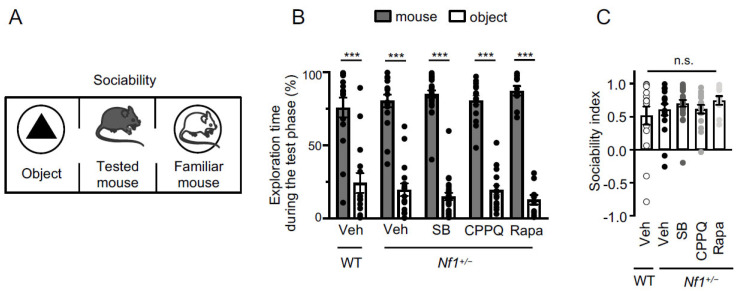

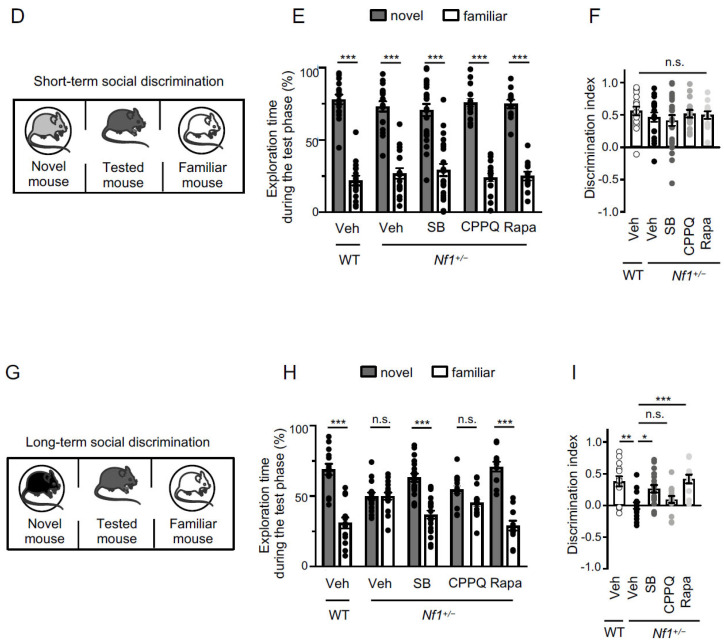
Impact of 5-HT_6_ receptor-operated mTOR signaling upon social cognition in *Nf1^+/−^* mice. Injections of vehicle, SB258585 (SB, 2.5 mg/kg, i.p.), CPPQ (2.5 mg/kg, i.p.) or rapamycin (Rapa, 10 mg/kg, i.p.) were performed 15 min before the habituation phase. (**A**). For assessing sociability, the lateral compartments of the three-chamber apparatus contain a wire cage with either an object or a mouse. (**B**). Exploration time (expressed in %) of the object and the congener by the tested mice. *** *p* < 0.001, significantly different from object; two-way ANOVA followed by Bonferroni’s test, with object or mouse and treatment as factors. (**C**). Sociability index in each condition (WT vehicle: *n* = 15, Nf1^+/−^ vehicle: *n* = 17, Nf1^+/−^ SB: *n* = 24, Nf1^+/−^ Rapa: *n* = 10, Nf1^+/−^ CPPQ: *n* = 16). (**D**). For assessing short-term social discrimination (5 min after the sociability phase), the lateral compartments of the three-chamber apparatus contain a wire cage with either the familiar mouse or a novel mouse (**E**)**.** Exploration time (expressed in %) of the novel and the familiar mouse by the tested mice. *** *p* < 0.001, significantly different from familiar mouse, two-way ANOVA followed by Bonferroni’s test, with novelty and treatment as factors. (**F**). Discrimination index in each condition (WT vehicle: *n* = 16, Nf1^+/−^ vehicle: *n* = 18, Nf1^+/−^ SB: *n* = 23, Nf1^+/−^ Rapa: *n* = 12, Nf1^+/−^ CPPQ: *n* = 15). (**G**). For assessing long-term social discrimination phase (24 h following the short-term social discrimination test), the lateral compartments of the three-chamber apparatus contain a wire cage with either the familiar mouse or a novel mouse. (**H**). Exploration time (expressed in %) of the novel and the familiar mouse by the tested mice. n.s. not significant, *** *p* < 0.001, significantly different from familiar mouse; two-way ANOVA followed by Bonferroni’s test, with novelty and treatment as factors. (**I**). Discrimination index in each condition (WT vehicle: *n* = 14, Nf1^+/−^ vehicle: *n* = 16, Nf1^+/−^ SB: *n* = 20, Nf1^+/−^ Rapa: *n* = 12, Nf1^+/−^ CPPQ: *n* = 15). n.s. not significant, * *p* < 0.05, ** *p* < 0.01, *** *p* < 0.001 vs. vehicle-treated *Nf1^+/−^* mice, Kruskal-Wallis followed by Dunn’s test.

We first investigated sociability of *Nf1^+/−^* mice by using the three-chamber social preference test (Figure 2A,D,G). After 10 min of habituation in the empty testing arena, mice were able to interact either with an inanimate object or an unfamiliar congener (sociability phase). *Nf1^+/−^* mice spent more time interacting with their congener than with the object (*p* ˂ 0.001, *n* = 17, two-way ANOVA followed by Bonferroni’s test, Table 1) and did not display sociability impairment, compared to WT mice (*p* > 0.05, Kruskal-Wallis followed by Dunn’s test, Figure 2B,C).

Then, we assessed preference for social novelty by performing the social discrimination test. After a 5-min retention interval, a novel congener was presented to the tested mouse, in addition to the familiar one (Figure 2D). Both vehicle-treated WT and *Nf1^+/−^* mice spent significantly more time interacting with the novel mouse, compared to the familiar one (*p* ˂ 0.001, *n* = 16 and *p* ˂ 0.001, *n* = 18, for vehicle-treated WT and *Nf1^+/−^* mice, respectively, two-way ANOVA followed by Bonferroni’s test, Figure 2E and Table 1). Both vehicle-treated WT and Nf1^+/−^ mice displayed a similar discrimination index (*p* > 0.05, Kruskal-Wallis followed by Dunn’s test, Figure 2F and Table 1). However, after a 24-h retention interval, social novelty discrimination was impaired in *Nf1^+/−^* mice, compared to WT mice (*p* < 0.001, Kruskal-Wallis followed by Dunn’s test, Figure 2H,I and Table 1).

We then explored whether blocking 5-HT_6_ receptor-operated mTOR signaling reverses long-term deficit in social discrimination observed in *Nf1^+/−^* mice. After a 24-h retention interval, *Nf1^+/−^* mice injected with SB258585 or rapamycin spent significantly more time interacting with the novel mouse, compared to the familiar one, in the social discrimination test (*p* ˂ 0.001, *n* = 20 and *p* ˂ 0.001, *n* = 12, for SB258585- and rapamycin-treated *Nf1^+/−^* mice, respectively, two-way ANOVA followed by Bonferroni’s test, Figure 2H,I and Table 1). However, *Nf1^+/−^* mice that received an acute injection (i.p.) of CPPQ showed similarly altered performances to vehicle-treated Nf1^+/−^ mice (*p* > 0.05, Kruskal-Wallis followed by Dunn’s test, Figure 2H,I and Table 1). 

Neither SB258585 nor CPPQ nor rapamycin administration modified the sociability performance (*p* > 0.05, Kruskal-Wallis followed by Dunn’s test, Figure 2B and Table 1) and short-term social memory of *Nf1^+/−^* mice (*p* > 0.05, Kruskal-Wallis followed by Dunn’s test, Figure 2C and Table 1). Likewise, SB258585, CPPQ or rapamycin administration to WT mice did not alter their sociability and their short- and long-term social memories (Supplementary Figure S2 and Supplementary Table S1).

### 2.3. Role of 5-HT_6_ Receptor-Operated mTOR Signaling in Associative Memory Deficit of Nf1^+/−^ Mice

**Figure 3:**
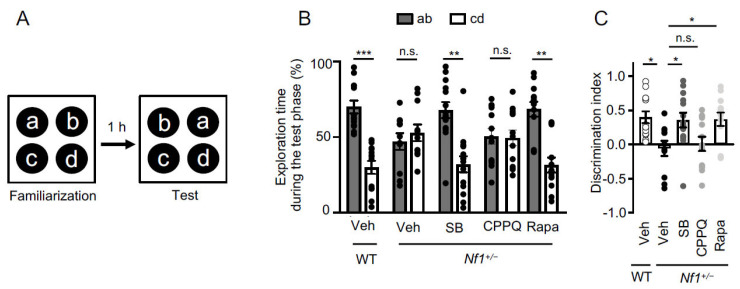
Impact of 5-HT_6_ receptor-operated mTOR signaling upon associative memory in *Nf1^+/−^* mice. Injections of vehicle, SB258585 (SB, 2.5 mg/kg, i.p.), CPPQ (2.5 mg/kg, i.p.) or rapamycin (Rapa, 10 mg/kg, i.p.) were performed 30 min before the familiarization phase. (**A**). Schema illustrating the procedure used for the object-in-place task. (**B**). The histograms represent the exploration time (expressed in %) of the different objects during the test phase. n.s. not significant, ** *p* < 0.01, *** *p* < 0.001, significantly different from non-swapped object; two-way ANOVA followed by Bonferroni’s test, with permutation and treatment as factors. (**C**). Discrimination index measured in each condition (WT vehicle: *n* = 13, Nf1^+/−^ vehicle: *n* = 11, Nf1^+/−^ SB: *n* = 14, Nf1^+/−^ Rapa: *n* = 13, Nf1^+/−^ CPPQ: *n* = 13). * *p* < 0.05 vs. vehicle-treated *Nf1^+/−^* mice, Kruskal-Wallis followed by Dunn’s test.

Impairments in executive function and mental flexibility have been reported in patients with NF1 [35]. We used the object-in-place task, a behavioral task assessing associative memory and involving mental flexibility and executive function [36,37]. In this test, mice must distinguish between four familiar objects in their original (familiarization phase) or a novel configuration (test phase). Memory retention was evaluated 1 h after the familiarization session. Vehicle-treated *Nf1^+/−^* mice did not discriminate between familiar and novel object configurations, as shown by the similar time they spent to explore the swapped object compared to the non-swapped objects during the test phase (*p* > 0.05, *n* = 11, two-way ANOVA followed by Bonferroni’s test, Figure 3 and Table 1), whereas vehicle-treated WT mice discriminated between familiar and novel object configurations (*n* = 13, *p* < 0.001, Kruskal-Wallis followed by Dunn’s test, Figure 3 and Table 1). Injection of SB258585 or rapamycin to *Nf1^+/−^* mice restored ability to discriminate between familiar and novel object configurations (*p* < 0.05 for *Nf1^+/−^* SB vs. *Nf1^+/−^* vehicle and *Nf1^+/−^* Rapa vs. *Nf1^+/−^* vehicle, Kruskal-Wallis followed by Dunn’s test, Figure 3 and Table 1). In contrast, CPPQ-injected *Nf1^+/−^* mice were still not able to discriminate novelty in the object-in-place test (*p* > 0.05 for *Nf1^+/−^* CPPQ vs. *Nf1^+/−^* vehicle, Figure 3 and Table 1). Injection of rapamycin or SB258585 or CPPQ to WT mice did not affect their performance in the object-in-place task (Supplementary Figure S3 and Supplementary Table S1).

## 3. Discussion

Loss-of-function mutations in the *NF1* gene encoding the Ras-GAP neurofibromin lead to Ras disinhibition and subsequent overactivation of Ras-dependent pathways, including the mTOR pathway. Consistently and corroborating previous findings [38,39,40], enhanced mTOR activity was observed concomitantly with reduced neurofibromin expression level in PFC of *Nf1^+/−^* mice, compared with WT mice. Cerebral mTOR can be activated by a large variety of extracellular signals. These include growth factors such as brain-derived neurotrophic factor, insulin, insulin-like growth factor 1 and vascular endothelial growth factor, which activate mTOR via their cognate tyrosine kinase receptors, and guidance molecules, such as reelin. A number of neurotransmitters including glutamate, dopamine, serotonin and endocannabinoids, are also known to activate the mTOR pathway in neurons through the stimulation of ionotropic or G protein-coupled receptors [41]. Among the 14 serotonin receptor subtypes, the 5-HT_6_ receptor physically interacts with and activates the mTOR complex 1 (mTORC1) in several brain regions upon agonist receptor stimulation. This receptor also physically interacts with neurofibromin, suggesting that neurofibromin might negatively regulate receptor-operated mTOR signaling and, conversely, that 5-HT_6_ receptors might contribute to the enhanced mTOR activity observed in *Nf1+/−* mouse brain. Consistent with this hypothesis, this non-physiological mTOR activation was abrogated by the peripheral administration of the specific 5-HT_6_ receptor antagonist SB258585, which thereby reproduced the effect of rapamycin treatment. 

A recent study reported elevated levels of serotonin in whole brain of *Nf1^+/−^* mice, compared to WT mice [42]. This elevation of cerebral serotonin levels might lead to persistent 5-HT_6_ receptor stimulation and subsequent overactivation of the mTOR pathway. On the other hand, 5-HT_6_ receptors are known to exhibit a high level of constitutive activity, not only in cell lines expressing high receptor densities, but also in the mouse brain [21]. Constitutive activity of 5-HT_6_ receptor is critically dependent of its dynamic association with protein partners, including neurofibromin [21,43]. Furthermore, we previously demonstrated that SB258585 behaves as a 5-HT_6_ receptor inverse agonist not only the toward canonical Gs signaling but also mTOR signaling [23]. Here, we show that in contrast to what was observed in SB258585-treated mice, administration of CPPQ, a well-characterized 5-HT_6_ receptor neutral antagonist, does not affect mTOR activity in PFC of *Nf1^+/−^*, suggesting that 5-HT_6_ receptor constitutive activity rather than agonist receptor stimulation contributes to mTOR overactivation in *Nf1^+/−^* mouse brain. 

Several preclinical studies using genetically engineered NF1 mouse models or established human tumor cell lines have demonstrated that mTOR overactivation underlies tumor proliferation in NF1, underpinning the therapeutic potential of rapamycin and its analogs [44,45,46]. Further supporting that mTOR inhibition may represent a relevant therapy for brain tumors in NF1, preliminary clinical studies confirmed unequivocal efficacy of rapamycin and its analogs to reduce the growth of plexiform neurofibroma [47] or low-grade glioma [48] observed in some patients with NF1. A large body of evidence also indicates a deleterious influence of aberrant mTOR signaling in the central nervous system upon cognition, in addition to its tumor growth promoting effects. Overactivation of mTOR has been involved in cognitive deficits observed in preclinical models of neurodevelopmental disorders, such as tuberous sclerosis, Fragile X syndrome and schizophrenia [20,49,50,51,52,53]. Likewise, non-physiological mTOR activation underlies cognitive deficits observed in preclinical models of acute cannabis consumption in adulthood and of cannabis abuse during adolescence [22,54] and those observed in neuropathic pain conditions [23,53,55]. Intriguingly, the role of mTOR overactivation in cognitive deficits associated with NF1 has so far not been investigated, while learning disabilities and deficits in attention, executive functions and perception skills are common complications seen in the majority of children carrying mutations in the *NF1* gene. In the present study, we show that *Nf1+/−* mice exhibit a normal sociability and short-term social memory, but alterations in long-term social memory and associative memory that are both abolished by an acute injection of rapamycin, indicating that deregulation of mTOR activity also plays a critical role in NF1-associated cognitive deficits. Notably, rapamycin did not affect social and associative memories in WT mice, indicating that only a non-physiological mTOR activation, such as that measured in PFC of *Nf1^+/−^* mice, affects these cognitive processes. 

Corroborating our results implicating constitutively active 5-HT_6_ receptors in the enhanced mTOR activity in PFC of *Nf1^+/−^* mice, we also demonstrated that the associated deficits of long-term social memory and associative memory are abolished by peripheral administration of the 5-HT_6_ receptor inverse agonist SB258585, but not a neutral antagonist (CPPQ). These results thus extend to NF1 previous observations indicating a critical influence of mTOR signaling under the control of 5-HT_6_ receptors, in cognitive deficits associated with pathological conditions of different etiologies, including preclinical models of schizophrenia or cannabis abuse during adolescence [20,22]. 

The mechanisms underlying the deleterious influence of 5-HT_6_ receptor-dependent mTOR overactivation upon cognitive functions in NF1 remain to be elucidated. A previous study showed that the specific deletion of neurofibromin in GABAergic neurons, but not pyramidal neurons, results in deficits in spatial learning (Morris water maze) that are caused by enhanced Erk activation in GABAergic neurons, increase in GABA release and subsequent deficits in hippocampal long-term potentiation LTP [56]. Together with the present results, these findings suggest that both enhanced Erk and mTOR activities contribute to cognitive impairment in NF1 and that deregulation of each pathway affects distinct cognitive functions. As Erk, mTOR finely tunes synaptic plasticity mechanisms such as LTP and long-term depression [41]. It is likely that overactivation of mTOR in PFC, one of the major brain structures involved in the control of cognition by 5-HT_6_ receptor, likewise leads to perturbations of synaptic transmission and synaptic plasticity in this brain region. Another study revealed the importance of deregulation of hyperpolarization-activated cyclic-nucleotide-gated channel 1 (HCN1, a neurofibromin-interacting protein) in the pathophysiology of NF1-associated cognitive deficits [57]. HCN1 is the predominant isoform of HCN channels, a family of voltage-gated channels that modulates neuronal excitability [58,59]. The contribution of HCN1 to perturbed cognition in NF1 is reminiscent of our observations in a mouse model of cannabis abuse during adolescence indicating that this treatment increases HCN1 activity and subsequently affects excitatory-inhibitory balance through a mechanism dependent of mTOR activation by 5-HT_6_ receptors [22]. Whether mTOR under the control of 5-HT_6_ receptors likewise affects HCN1 activity and excitatory-inhibitory balance in *Nf1^+/−^* mice remains to be explored. 

In conclusion, the present study provides a proof of principle that 5-HT_6_ receptor inverse agonists already in clinical development as symptomatic treatments to reduce cognitive decline in dementia such as Alzheimer’s disease or psychoses such as schizophrenia, might be repurposed as first-line treatments to alleviate alterations of cognitive functions in NF1 patients before the appearance of malignancies. Such a strategy would certainly be more relevant than direct mTOR blockade by pharmacological inhibitors at early disease stages, as 5-HT_6_ receptor inverse agonists are well tolerated and will thus not reproduce the severe side effects induced by mTOR inhibitors and related to their immunosuppressant actions. Given the influence of mTOR in the growth of benign and malignant cerebral tumors associated with NF1 and the role of constitutively active 5-HT_6_ receptors in the enhanced cerebral mTOR activity in *Nf1^+/−^* mice, the impact of 5-HT_6_ receptor inverse agonists on NF1-associated tumors certainly warrants further exploration.

## 4. Material and Methods

### 4.1. Animals

*Nf1^+/−^* mice (Brannan et al., 1994) were F1 hybrids from a cross between C57BL/6J and 129S2/SvPasCrl mice (Charles River France, L’Arbresle, France). Two-month-old mice from both sexes were used. All experiments used wild type littermates as controls. Mice were housed under standardized conditions with a 12-h light/dark cycle, stable temperature (22 ± 1 °C), controlled humidity (55 ± 10%) and free access to food and water. Animal husbandry and experimental procedures were performed in compliance with the animal use and care guidelines of the University of Montpellier, the French Agriculture Ministry and the European Council Directive (86/609/EEC). 

### 4.2. Drug Administration

4-Iodo-N-[4-methoxy-3-(4-methylpiperazin-1-yl)-phenyl]benzenesulfonamide (SB258585) was purchased from Sigma-Aldrich (St Quentin Fallavier, France) and rapamycin from LC Laboratories (Woburn, MA, USA). CPPQ was synthetized as previously described [33,34]. SB258585 (injected at 2.5 mg/kg, i.p.), rapamycin (injected at 10 mg/kg, i.p.) and CPPQ (injected at 2.5 mg/kg, i.p.) were dissolved in 5% DMSO/5% Tween 80 in NaCl. 

### 4.3. Behavioral Tests

The sample size was assessed using the G power software. It was set with the mean value of 12, based on our previous results assuming a significance level of 5% and a power of 80%. 

#### 4.3.1. Social Behavior Tests

Testing was carried out in a rectangular, three-chamber box with dividing walls made of clear Plexiglas and an open middle section, which allows free access to each chamber [60]. The social interaction test was conducted as previously described [30]. Briefly, mice were extensively handled one week before behavioral analysis. Over two days, four 10-min sessions were conducted. First, a habituation session was conducted in which the tested mice were placed for 10 min in the middle compartment of the three-chamber device without the walls between the compartments, to allow free access to the three compartments and the empty cylindric containment cages placed in the lateral compartments. After a 5-min retention interval, a social interaction session was conducted for 10 min (mouse of same age and sex as tested mice or inanimate object placed in the containment cages). Five min after the social interaction session, a short-term social discrimination test was conducted for 10 min (familiar mouse and a novel mouse of same age and sex as tested mice, in the containment cages). Following the short-term social discrimination session, the «novel» mouse was removed from the apparatus, and the tested mouse was allowed to interact with the «familiar» mouse for an additional 45 min. A long-term social discrimination session (familiar mouse and a novel mouse of same age and sex as tested mice, in the containment cages) was carried out 24 h after the short-term social discrimination session. Duration of direct contacts between the tested mouse and the cage housing the conspecific or the object was recorded for 3 min. The experiments were video-recorded and exploration times (nose in contact or sniffing at < 1 cm) were measured by a blinded observer. Sociability (exploration time of the mouse—exploration time of the object/total exploration time) and discrimination indexes (exploration time of the novel mouse—exploration time of the familiar mouse/total exploration time) and percentages of exploration were compared between groups. Drugs were administered 15 min before the habituation session on day 1 and 45 min before the long-term social discrimination on day 2. Animals that did not receive drugs were injected with an equivalent volume of vehicle. Animals that stayed only in the central chamber during one of the 3 steps of the test were excluded.

#### 4.3.2. Object-in-Place Task

Testing was carried out in an arena (50-cm width, 50-cm length, 50-cm height) placed in a dimly lit room with clearly visible contextual cues (black on white patterns) on the surrounding walls. Mice were habituated to the arena on day 1 for 10 min. On day 2, mice had a 10-min familiarization session with four different objects presented and placed in the four corners of the arena. Mice were transferred back to the home cage during 1-h retention interval before a 3-min training session with two objects swapped. The objects were plastic toys (3-cm width, 3-cm length, 5-cm height) and were cleaned with 20% ethanol between sessions. The experiments were video-recorded and exploration times (nose in contact with or sniffing objects at < 1 cm) were measured by a blinded observer. Discrimination indexes (exploration time of novel object—exploration time of familiar object)/total object exploration time) were compared between groups. Mice with total exploration time of less than 3 s in the test session were excluded.

### 4.4. Western Blotting

Mice were killed by cervical dislocation and the heads were immersed in liquid nitrogen for 4 s. Brains were removed and the prefrontal cortex was rapidly dissected and homogenized in lysis buffer (Tris-HCl 50 mM pH7.5, NaCl 150 mM, sucrose 0.27 M, EDTA 2 mM, EGTA 2 mM, SDS 2% *w*/*v*, NaF 50 mM, Na orthovanadate 1 mM, Na pyrophosphate 5 mM, β-glycerophosphate, 25 mM) using a potter, sonicated and then centrifuged at 10,000× *g* for 5 min. The supernatant was used for protein analysis.

Equal amounts of protein (30 µg) from each sample were resolved onto 4–15% polyacrylamide gels (Mini protean TGX stain-free gels, Bio-Rad, Le Relecq Kerhuon, France Cat#456-8084). Proteins were transferred to nitrocellulose membranes (TransBlot Turbo Midi nitrocellulose, Bio-Rad). After 1 h in the blocking solution (Tris–HCl, 50 mM, pH 7.5; NaCl, 200 mM; Tween-20, 0.1%, and skimmed dried milk, 5%), membranes were immunoblotted with primary antibodies: anti-neurofibromin (1:500, Santa-Cruz, Heidelberg, Germany, SC-376886), anti-phospho-Thr^421^/Ser^424^-P70S6K (1:1000, Cell Signaling, Leiden, Holland, ref. 9204), anti-P70S6K (1:500, Cell Signaling, ref. 9202), mouse anti-pan Actin (1:2000, Fisher Scientific, Illkirch, France ref. MS-1295-B) and then with either anti-mouse or anti-rabbit horseradish peroxidase-conjugated secondary antibodies (1:5000, GE Healthcare). Immunoreactivity was detected with an enhanced chemiluminescence method using a Chemidoc Touch imaging system (Bio-Rad). Immunoreactive bands were quantified by densitometry using the Image J software. For p70S6K phosphorylation state analysis, the amount of phosphoprotein (immunoreactive signal obtained with the anti-phospho-Thr^421^/Ser^424^-P70S6K antibody) was normalized to the amount of total p70S6K (immunoreactive signal obtained with the antibody recognizing P70S6K independently of its phosphorylation state) detected in the sample. Neurofibromin level in the samples was normalized to actin level. 

### 4.5. Statistics

Data were analyzed using the GraphPad Prism software (v.9, GraphPad Software, San Diego, CA, USA). Biochemistry experiments were analyzed by one-way ANOVA followed by Dunnett’s test. Discrimination indexes of behavioral experiments were analyzed by Kruskal-Wallis followed by Dunn’s multiple comparisons test while exploration times of object vs. mouse, familiar vs. novel mice and swapped vs. non-swapped object were analyzed by two-way ANOVA, followed by Bonferroni’s test for multiple comparisons.

## Figures and Tables

**Table 1:** Discrimination Indexes and exploration time (in percent) obtained for each behavioral test performed.

**Behavioral Test**	**Experimental Conditions**	**Discrimination Index** **(mean ± sem)**
Sociability	WT + Vehicle	0.52 ± 0.14
	Nf1^+/−^ + Vehicle	0.61 ± 0.08
	Nf1^+/−^ + SB258585	0.70 ± 0.05
	Nf1^+/−^ + CPPQ	0.61 ± 0.06
	Nf1^+/−^ + Rapamacyn	0.75 ± 0.07
Short-term memory	WT + Vehicle	0.56 ± 0.07
	Nf1^+/−^ + Vehicle	0.46 ± 0.07
	Nf1^+/−^ + SB258585	0.42 ± 0.08
	Nf1^+/−^ + CPPQ	0.52 ± 0.06
	Nf1^+/−^ + Rapamacyn	0.50 ± 0.06
Long-term memory	WT + Vehicle	0.38 ± 0.08
	Nf1^+/−^ + Vehicle	0.00 ± 0.05
	Nf1^+/−^ + SB258585	0.26 ± 0.06
	Nf1^+/−^ + CPPQ	0.09 ± 0.05
	Nf1^+/−^ + Rapamacyn	0.42 ± 0.07
Object-in-place	WT + Vehicle	0.40 ± 0.09
	Nf1^+/−^ + Vehicle	-0.06 ± 0.11
	Nf1^+/−^ + SB258585	0.36 ± 0.11
	Nf1^+/−^ + CPPQ	0.01 ± 0.10
	Nf1^+/−^ + Rapamacyn	0.37 ± 0.10
**Behavioral test**	**Experimental Conditions**	**Exploration Time in %** **(mean ± sem)**
Sociability	WT + Vehicle—mouse	75.81 ± 6.79
	WT + Vehicle—object	24.19 ± 6.79
	Nf1^+/−^ + Vehicle—mouse	80.42 ± 4.27
	Nf1^+/−^ + Vehicle—object	19.58 ± 4.27
	Nf1^+/−^ + SB—mouse	85.10 ± 2.54
	Nf1^+/−^ + SB—object	14.90 ± 2.54
	Nf1^+/−^ + CPPQ—mouse	80.74 ± 3.20
	Nf1^+/−^ + CPPQ—object	19.26 ± 3.20
	Nf1^+/−^ + Rapa—mouse	87.27 ± 3.27
	Nf1^+/−^ + Rapa—object	12.73 ± 3.27
Short-term memory	WT + Vehicle—novel	78.12 ± 3.33
	WT + Vehicle—familiar	21.88 ± 3.33
	Nf1^+/−^ + Vehicle—novel	73.24 ± 3.61
	Nf1^+/−^ + Vehicle—familiar	26.76 ± 3.61
	Nf1^+/−^ + SB—novel	70.78 ± 4.16
	Nf1^+/−^ + SB—familiar	29.22 ± 4.16
	Nf1^+/−^ + CPPQ—novel	75.98 ± 2.95
	Nf1^+/−^ + CPPQ—familiar	24.02 ± 2.95
	Nf1^+/−^ + Rapa—novel	74.97 ± 2.99
	Nf1^+/−^ + Rapa—familiar	25.03 ± 2.99
Long-term memory	WT + Vehicle—novel	69.01 ± 3.96
	WT + Vehicle—familiar	30.99 ± 3.96
	Nf1^+/−^ + Vehicle—novel	49.96 ± 2.58
	Nf1^+/−^ + Vehicle—familiar	50.02 ± 2.58
	Nf1^+/−^ + SB—novel	63.18 ± 2.89
	Nf1^+/−^ + SB—familiar	36.82 ± 2.88
	Nf1^+/−^ + CPPQ—novel	54.72 ± 2.74
	Nf1^+/−^ + CPPQ—familiar	45.28 ± 2.74
	Nf1^+/−^ + Rapa—novel	70.85 ± 3.45
	Nf1^+/−^ + Rapa—familiar	29.15 ± 3.45
Object-in-place	WT + Vehicle—« ab »	69.98 ± 4.31
	WT + Vehicle—« cd »	30.02 ± 4.31
	Nf1^+/−^ + Vehicle—« ab »	47.13 ± 5.49
	Nf1^+/−^ + Vehicle—« cd »	52.87 ± 5.49
	Nf1^+/−^ + SB—« ab »	67.92 ± 5.32
	Nf1^+/−^ + SB—« cd »	32.08 ± 5.32
	Nf1^+/−^ + CPPQ—« ab »	50.54 ± 5.18
	Nf1^+/−^ + CPPQ—« cd »	49.46 ± 5.18
	Nf1^+/−^ + Rapa—« ab »	68.47 ± 4.89
	Nf1^+/−^ + Rapa—« cd »	31.53 ± 4.89

## Data Availability

All the data are illustrated in the figures and in the supplementary data, except for video recordings of behavioral experiments which are available on request from the corresponding authors.

## Supplementary Materials

The following are available online at https://www.mdpi.com/1422-0067/22/18/10178/s1.
