# Supplementary material for: Blockade of Serotonin 5-HT6 Receptor Constitutive Activity Alleviates Cognitive Deficits in a Preclinical Model of Neurofibromatosis Type 1
Source: Int J Mol Sci. 2021 Sep 21;22(18):10178. doi: 10.3390/ijms221810178 (PMC8467191; doi:10.3390/ijms221810178)
Supplement: Supplementary file 1 [file ijms-22-10178-s001.zip › ijms-1318032-supplementary.pdf]

Blockade of Serotonin 5-HT$_6$ Receptor Constitutive Activity Alleviates Cognitive Deficits in a Preclinical Model of Neurofibromatosis Type 1

Supplementary Figures, Legends and Table. Doucet E. et al. IJMS 2021

Supplementary Figure S1. Original Western blots used to quantify the immunoreactive bands. Representative bands illustrated on Figure 1 are framed in red. All bands used for the quantification are outlined in green. **A.** Western blot assessing neurofibromin and actin expression in PFC of adult WT (n=3) and *Nf1*$^{+/-}$ mice (n=5). **B.** Western blots assessing p70S6K phosphorylation at Thr421-Ser424 and p70S6K expression in PFC of adult WT (n=3) and *Nf1*$^{+/-}$ mice (n=5). **C.** Western blots assessing p70S6K phosphorylation at Thr421-Ser424 and p70S6K expression in PFC of *Nf1*$^{+/-}$ mice injected with either vehicle (n=3) or Rapamycin (Rapa, 10 mg/kg, n=3), or SB258585 (SB, 2.5 mg/kg, n=3)

Supplementary Figure S2. Blockade of the 5-HT$_6$ receptor-mTOR pathway does not affect sociability and short-term and long-term social discrimination in wildtype mice

WT mice were injected with either vehicle, or SB258585 (SB, 2.5 mg/kg, i.p.), or CPPQ (2.5 mg/kg, i.p.) or rapamycin (Rapa, 10 mg/kg, i.p.) 15 min before the habituation phase. **A.** Schema illustrating the procedure used for assessing sociability of WT mice. **B.** Exploration time (expressed in %) of the object and the congener by the tested mice. *** $p < 0.001$, significantly different from object; two-way ANOVA followed by Bonferroni's test, with object and treatment as factors. **C.** Sociability index in each condition (vehicle: n = 15, SB: n = 12, Rapa: n = 14, CPPQ: n = 15). **D.** Schema illustrating the procedure used for assessing short-term social discrimination. **E.** Exploration time (expressed in %) of the novel and the familiar mouse by the tested mice. *** $p < 0.001$, significantly different from familiar mouse; two-way ANOVA followed by Bonferroni's test, with novelty and treatment as factors. **F.** Discrimination index in the corresponding conditions (vehicle: n = 16, SB: n = 13, Rapa: n =

12, CPPQ: n = 15). **G.** Schema illustrating the procedure used for assessing long-term social discrimination. **H.** Exploration time (expressed in %) of the novel and the familiar mouse by the tested mice. ** $p<0.01$, *** $p<0.001$, significantly different from familiar mouse; two-way ANOVA followed by Bonferroni's test, with novelty and treatment as factors. **I.** Discrimination index in the corresponding conditions (vehicle: n = 14, SB: n = 13, Rapa: n = 13, CPPQ: n = 17). n.s. non-significant *vs.* vehicle-injected WT mice, Kruskal-Wallis followed by Dunn's test.

Supplementary Figure S3. Blockade of the 5-HT$_6$ receptor-mTOR pathway does not affect associative memory in WT mice

WT mice were injected with either vehicle, or SB258585 (SB, 2.5 mg/kg, i.p.), CPPQ (2.5 mg/kg, i.p.) or rapamycin (Rapa, 10 mg/kg, i.p.) 30 min before the familiarization phase. **A.** Exploration time (expressed in %) of the different objects during the test phase. * $p<0.05$, *** $p < 0.001$ significantly different from non-swapped object; two-way ANOVA followed by Bonferroni's test, with permutation and treatment as factors. **B.** Discrimination index measured in each condition. n.s. non-significant *vs.* vehicle-injected WT mice, Kruskal-Wallis followed by Dunn's test.

Supplementary Table S1. Discrimination Indexes and exploration time (in percent) for each behavioral test performed.

Supp. Fig. S1

A. Western blots corresponding to Figure 1A

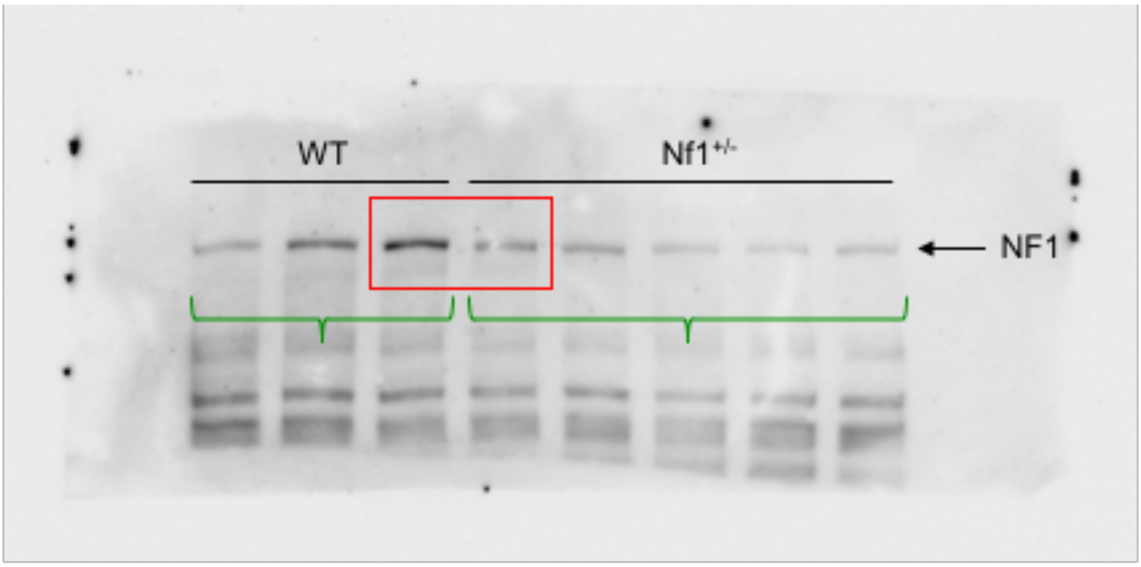

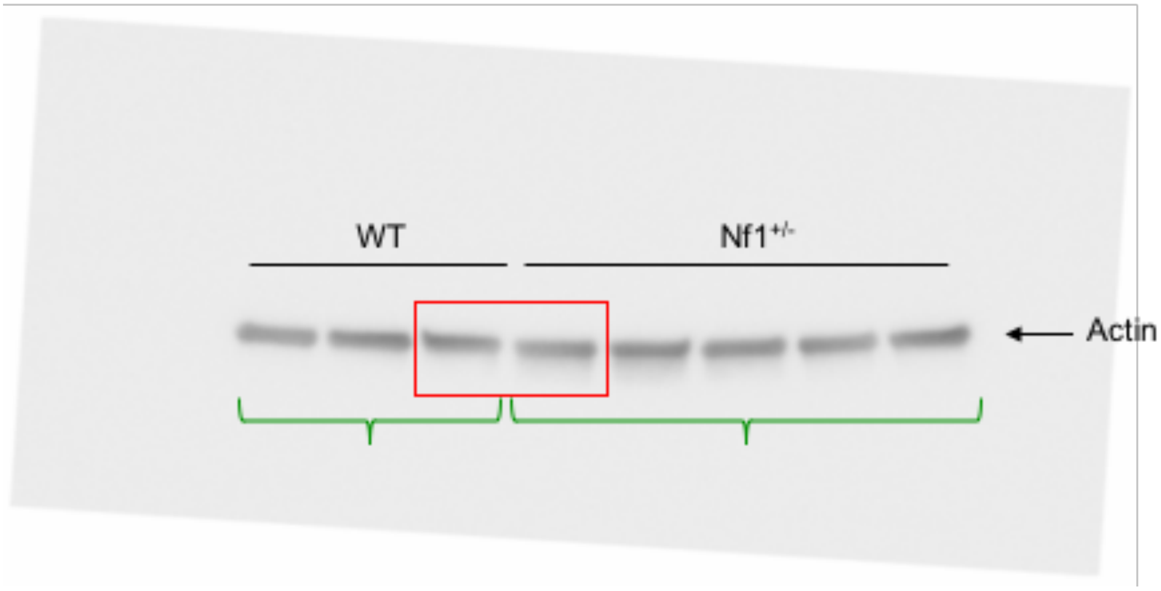

B. Western blots corresponding to Figure 1B

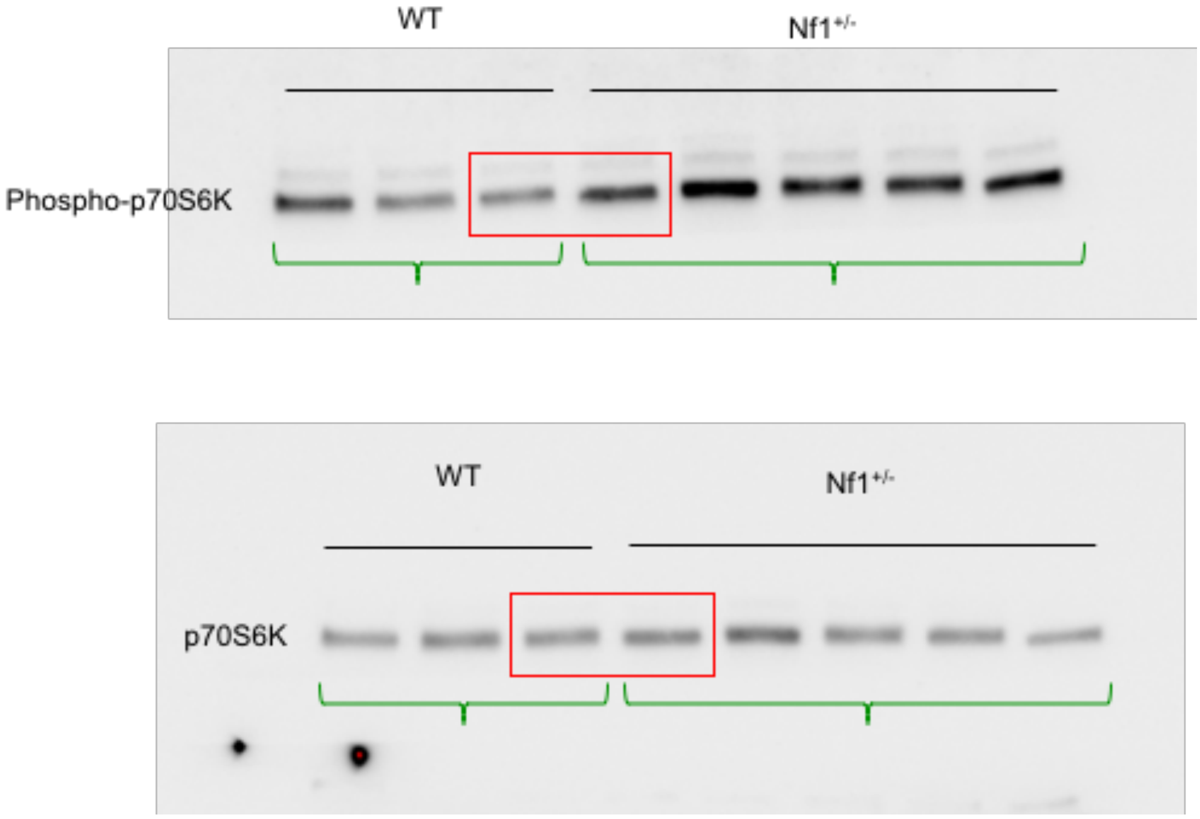

C. Western blots corresponding to Figure 1C

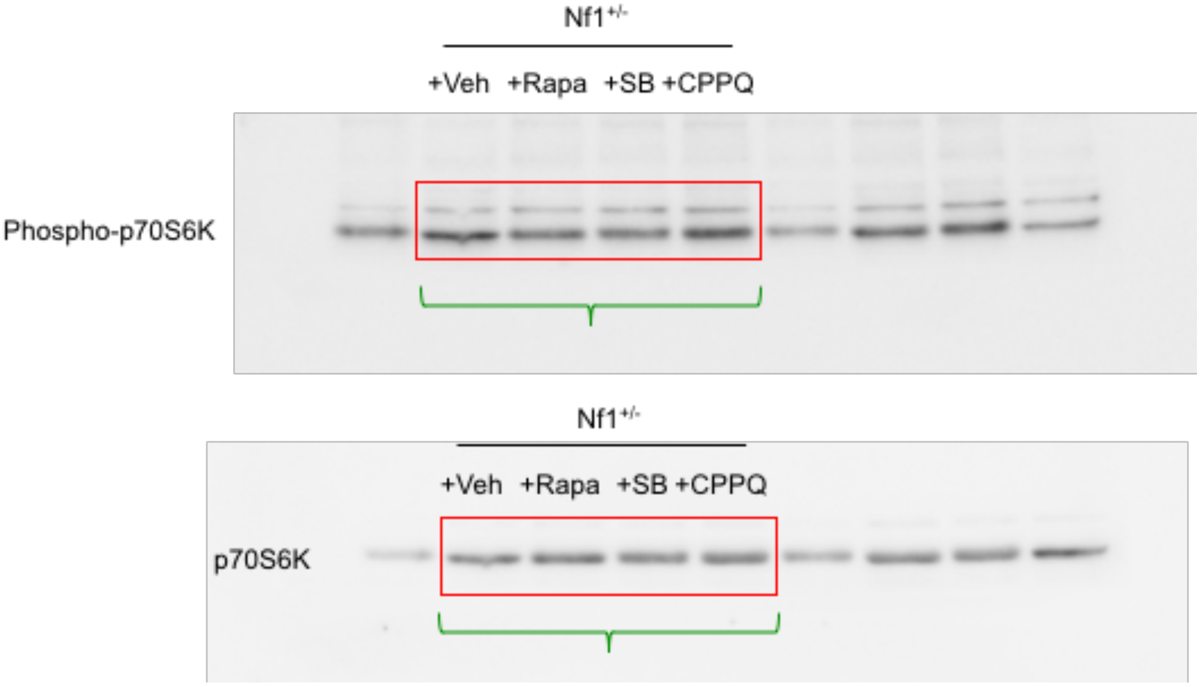

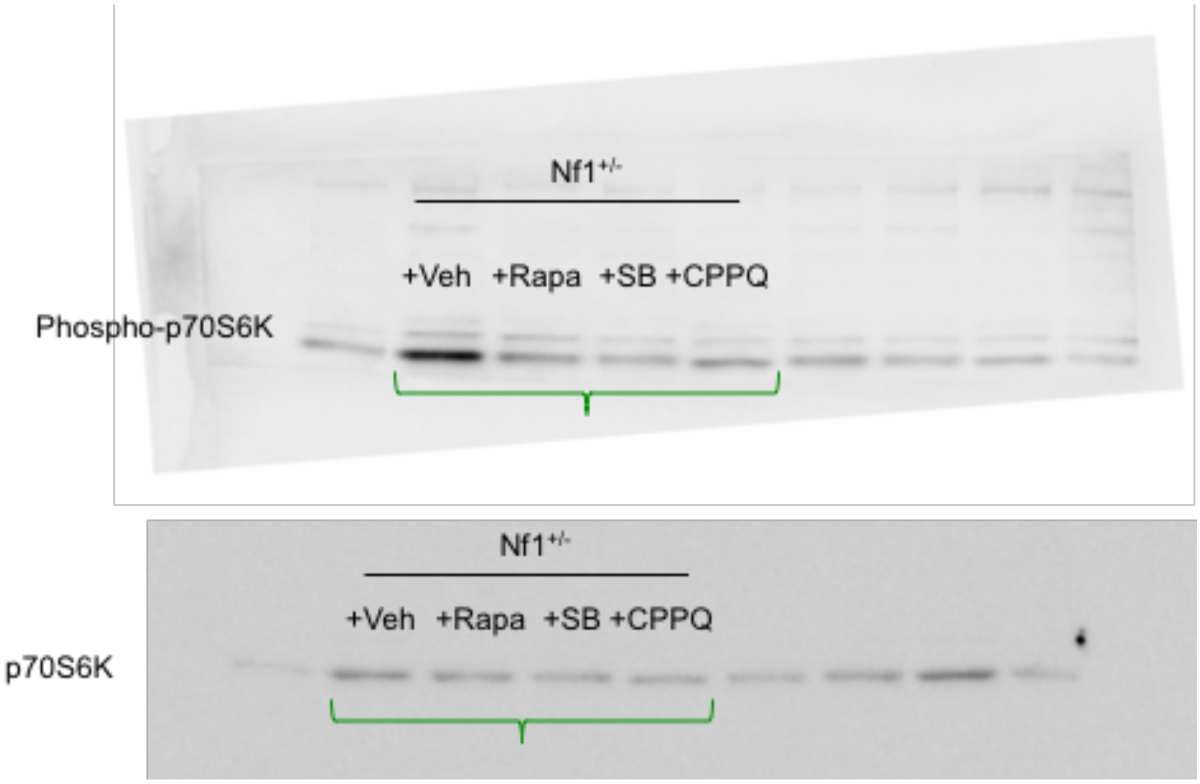

Nf1+/-
+Veh +Rapa +SB +CPPQ
Phospho-p70S6K
Nf1+/-
+Veh +Rapa +SB +CPPQ
p70S6K

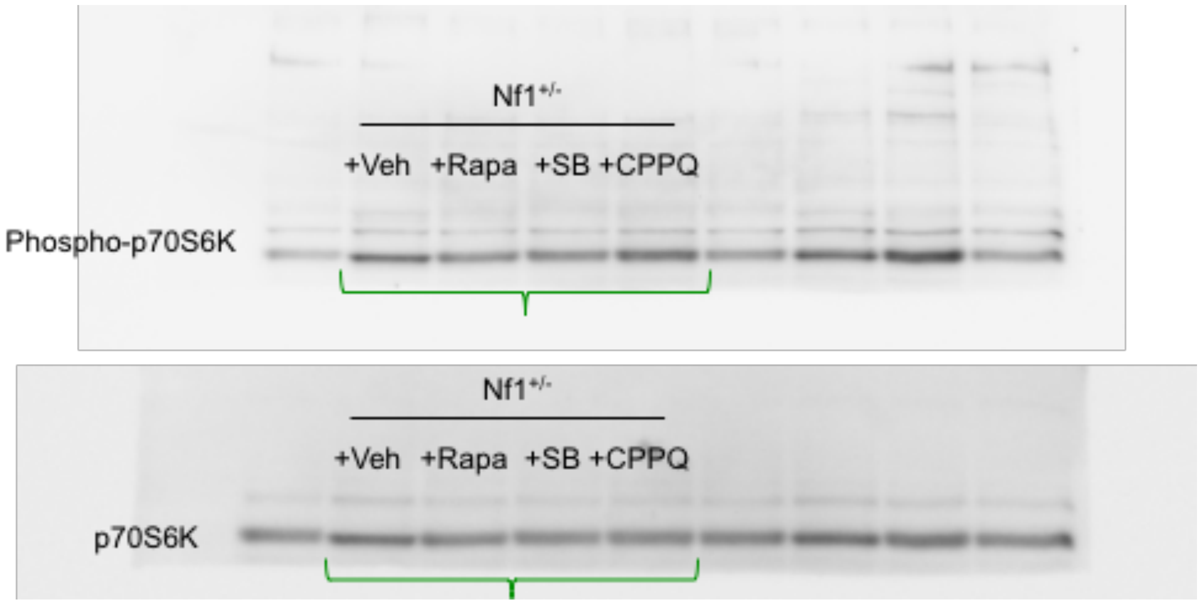

Nf1+/-
+Veh +Rapa +SB +CPPQ
Phospho-p70S6K
Nf1+/-
+Veh +Rapa +SB +CPPQ
p70S6K

Supp Fig S2

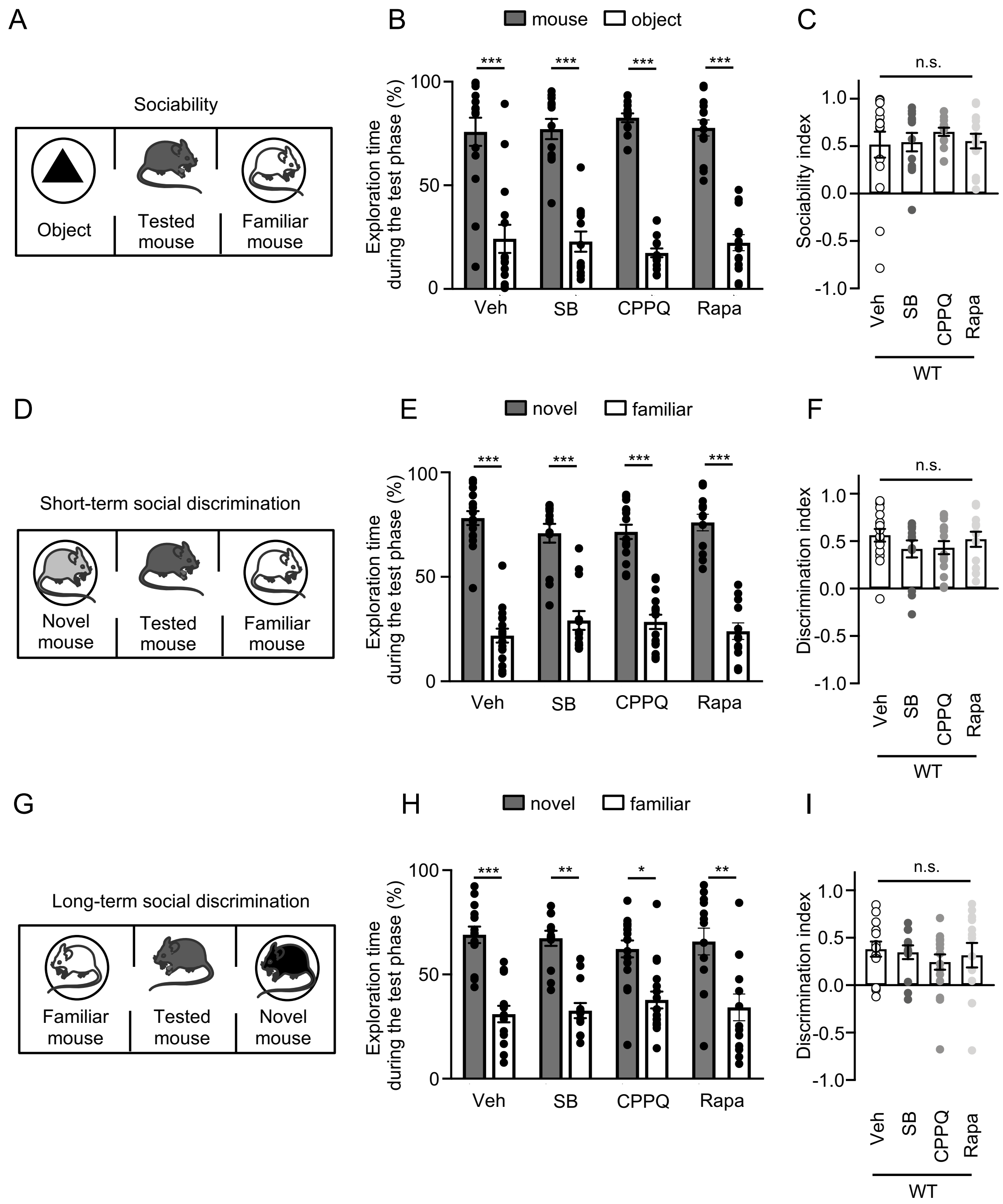

A
Sociability
Object
Tested mouse
Familiar mouse
B
mouse
object
Exploration time during the test phase (%)
***
Veh
SB
CPPQ
Rapa
C
n.s.
Sociability index
Veh
SB
CPPQ
Rapa
WT
D
Short-term social discrimination
Novel mouse
Tested mouse
Familiar mouse
E
novel
familiar
Exploration time during the test phase (%)
F
n.s.
Discrimination index
WT
G
Long-term social discrimination
Familiar mouse
Tested mouse
Novel mouse
H
novel
familiar
Exploration time during the test phase (%)
***
**
*
I
n.s.
Discrimination index
WT

Supp Fig S3

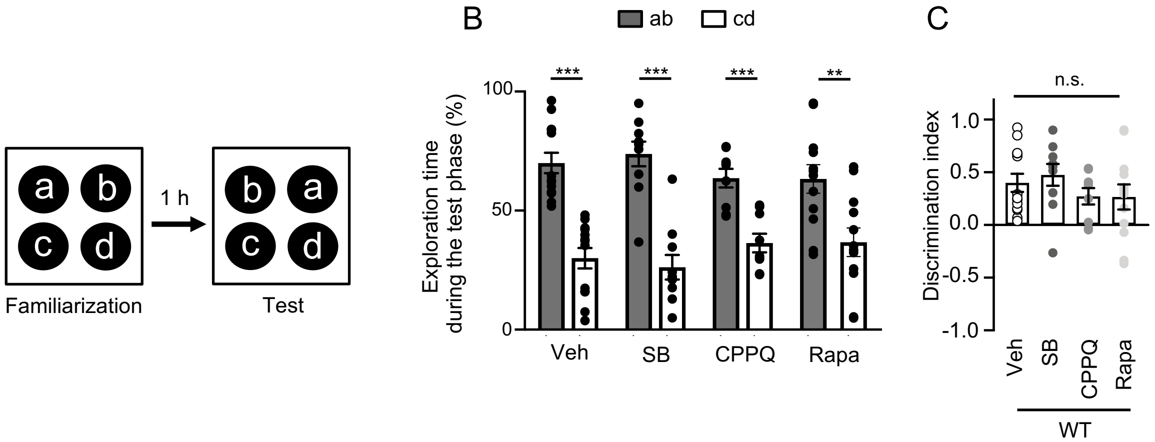

a
b
c
d
1 h
b
a
c
d
Familiarization
Test
B
ab
cd
Exploration time during the test phase (%)
100
50
0
***
***
***
**
Veh
SB
CPPQ
Rapa
C
n.s.
Discrimination index
1.0
0.5
0.0
-0.5
-1.0
Veh
SB
CPPQ
Rapa
WT

Supp. Table S1

Behavioral test	Experimental conditions	Discrimination index (mean±sem)
Sociability	WT + Vehicle	0.52 ± 0.14
	WT + SB258585	0.54 ± 0.10
	WT + CPPQ	0.65 ± 0.04
	WT + Rapamacyn	0.55 ± 0.08
Short-term memory	WT + Vehicle	0.56 ± 0.07
	WT + SB258585	0.48 ± 0.09
	WT + CPPQ	0.43 ± 0.07
	WT + Rapamacyn	0.52 ± 0.08
Long-term memory	WT + Vehicle	0.38 ± 0.08
	WT + SB258585	0.35 ± 0.07
	WT + CPPQ	0.25 ± 0.08
	WT + Rapamacyn	0.32 ± 0.13
Object-in-place	WT + Vehicle	0.40 ± 0.09
	WT + SB258585	0.48 ± 0.10
	WT + CPPQ	0.27 ± 0.08
	WT + Rapamacyn	0.27 ± 0.12
**Behavioral test**	**Experimental conditions**	**Exploration time in % (mean±sem)**
Sociability	WT + Vehicle - novel	75.81 ± 6.79
	WT + Vehicle - familiar	24.19 ± 6.79
	WT + SB - novel	77.15 ± 4.88
	WT + SB - familiar	22.85 ± 4.88
	WT + CPPQ- novel	82.59 ± 2.14
	WT + CPPQ - familiar	17.41 ± 2.14
	WT + Rapa - novel	77.69 ± 3.87
	WT + Rapa - familiar	22.31 ± 3.87
Short-term memory	WT + Vehicle - novel	78.12 ± 3.33
	WT + Vehicle - familiar	21.88 ± 3.33
	WT + SB - novel	70.89 ± 4.52
	WT + SB - familiar	29.11 ± 4.52
	WT + CPPQ- novel	71.56 ± 3.47
	WT + CPPQ - familiar	28.44 ± 3.47
	WT + Rapa - novel	76.00 ± 3.97
	$NF^{+/-}$ + Rapa - familiar	24.00 ± 3.97
Long-term memory	WT + Vehicle - novel	69.01 ± 3.96
	WT + Vehicle - familiar	30.99 ± 3.96
	WT + SB - novel	67.35 ± 3.61
	WT + SB - familiar	32.65 ± 3.61
	WT + CPPQ- novel	62.24 ± 4.03
	WT + CPPQ - familiar	37.76 ± 4.03
	WT + Rapa - novel	65.80 ± 6.44
	WT + Rapa - familiar	34.20 ± 6.44

Object-in-place	WT + Vehicle – « ab »	69.98 ± 4.31
	WT + Vehicle – « cd »	30.02 ± 4.31
	WT + SB - « ab »	73.77 ± 5.17
	WT + SB - « cd »	26.23 ± 5.17
	WT + CPPQ- « ab »	63.60 ± 3.90
	WT + CPPQ - « cd »	36.40 ± 3.90
	WT + Rapa - « ab »	63.29 ± 5.96
	WT + Rapa - « cd »	36.71 ± 5.96
